# The role of shear stress in Blood-Brain Barrier endothelial physiology

**DOI:** 10.1186/1471-2202-12-40

**Published:** 2011-05-11

**Authors:** Luca Cucullo, Mohammed Hossain, Vikram Puvenna, Nicola Marchi, Damir Janigro

**Affiliations:** 1 Cerebrovascular Research, Lerner Research Institute, Cleveland Clinic, Cleveland, OH 44195 USA; 2 Dept. of Cell Biology, Cleveland Clinic, Cleveland, OH 44195 USA; 3 Department of Molecular Medicine, Cleveland Clinic Lerner College of Medicine, Cleveland, OH 44195 USA

**Keywords:** Cerebral blood flow, Shear stress, Cell Cycle, Alternative, In vitro, Inflammation

## Abstract

**Background:**

One of the most important and often neglected physiological stimuli contributing to the differentiation of vascular endothelial cells (ECs) into a blood-brain barrier (BBB) phenotype is shear stress (SS). With the use of a well established humanized dynamic *in vitro *BBB model and cDNA microarrays, we have profiled the effect of SS in the induction/suppression of ECs genes and related functions.

**Results:**

Specifically, we found a significant upregulation of tight and adherens junctions proteins and genes. Trans-endothelial electrical resistance (TEER) and permeability measurements to know substances have shown that SS promoted the formation of a tight and highly selective BBB. SS also increased the RNA level of multidrug resistance transporters, ion channels, and several p450 enzymes. The RNA level of a number of specialized carrier-mediated transport systems (e.g., glucose, monocarboxylic acid, etc.) was also upregulated.

RNA levels of modulatory enzymes of the glycolytic pathway (e.g., lactate dehydrogenase) were downregulated by SS while those involved in the Krebs cycle (e.g., lactate and other dehydrogenases) were upregulated. Measurements of glucose consumption versus lactate production showed that SS negatively modulated the glycolytic bioenergetic pathways of glucose metabolism in favor of the more efficient aerobic respiration. BBB ECs are responsive to inflammatory stimuli. Our data showed that SS increased the RNA levels of integrins and vascular adhesion molecules. SS also inhibited endothelial cell cycle via regulation of BTG family proteins encoding genes. This was paralleled by significant increase in the cytoskeletal protein content while that of membrane, cytosol, and nuclear sub-cellular fractions decreased. Furthermore, analysis of 2D gel electrophoresis (which allows identifying a large number of proteins per sample) of EC proteins extracted from membrane sub-cellular endothelial fractions showed that SS increased the expression levels of tight junction proteins. In addition, regulatory enzymes of the Krebb's cycle (aerobic glucose metabolism) were also upregulated. Furthermore, the expression pattern of key protein regulators of the cell cycle and parallel gene array data supported a cell proliferation inhibitory role for SS.

**Conclusions:**

Genomic and proteomic analyses are currently used to examine BBB function in healthy and diseased brain and characterize this dynamic interface. In this study we showed that SS plays a key role in promoting the differentiation of vascular endothelial cells into a truly BBB phenotype. SS affected multiple aspect of the endothelial physiology spanning from tight junctions formation to cell division as well as the expression of multidrug resistance transporters. BBB dysfunction has been observed in many neurological diseases, but the causes are generally unknown. Our study provides essential insights to understand the role played by SS in the BBB formation and maintenance.

## Background

The blood-brain barrier is a dynamic interface between the blood and the central nervous system (CNS), that controls the influx and efflux of biological substances needed for the brain metabolic processes, as well as for neuronal function. Therefore the functional and structural integrity of the BBB is vital to maintain the homeostasis of the brain microenvironment.

At the cellular level, the BBB consists of microvascular endothelial cells (EC) lining the brain microvessels together with the closely associated astrocytic end-feet processes [1]. The microcapillary endothelium is characterized by the presence of tight junctions, lack of fenestrations, and minimal pinocytotic vesicles. In particular, tight junctions between the cerebral endothelial cells form a diffusion barrier, which selectively excludes most blood-borne substances from entering the brain, protecting it from systemic influences mediated by substances of all size or polar molecules such as water soluble compounds (electrolytes). Transport for nutrients (as well as other biologically important substances) from the peripheral circulation into brain parenchyma requires translocation through the capillary endothelium by specialized carrier-mediated transport systems. Membrane localization of these enzymes is indicative of the polarity of the endothelial functions in the control of the blood-brain interface [2]. The BBB endothelial cytoplasm is richly endowed with enzymes, including monoamine oxidase, acid and alkaline phosphatases, p450 enzymes [3] and is also characterized by very high density of mitochondria denoting high metabolic activity [4]. Furthermore, the cellular membrane hosts a variety of adhesion molecules and integrins that allow for the interaction with the host immune system when activated by pro-inflammatory stimuli [5].

This plethora of highly specialized functions is indicative of a significant level of differentiation that sets apart the BBB endothelium from that of other vascular beds. While the physiological environment is certainly responsible for the differentiation of these endothelial cells into a BBB phenotype, the mechanisms involved are not fully understood. The surrounding cellular elements (e.g., astrocytes) by means of trophic stimuli (some still unknown) are crucially important for the EC differentiation however, there is an underestimated and poorly understood mechanical stimuli that also plays a major role in this process, such as the exposure to shear stress (SS). SS is a tangential force generated by flow across the apical surface of vascular endothelium. In this study we show that SS affects endothelial cell by modulating the induction/suppression of genes, which impact the development of BBB properties and functions.

## Results

### Shear stress promoted BBB tightness

**Figure 1:**
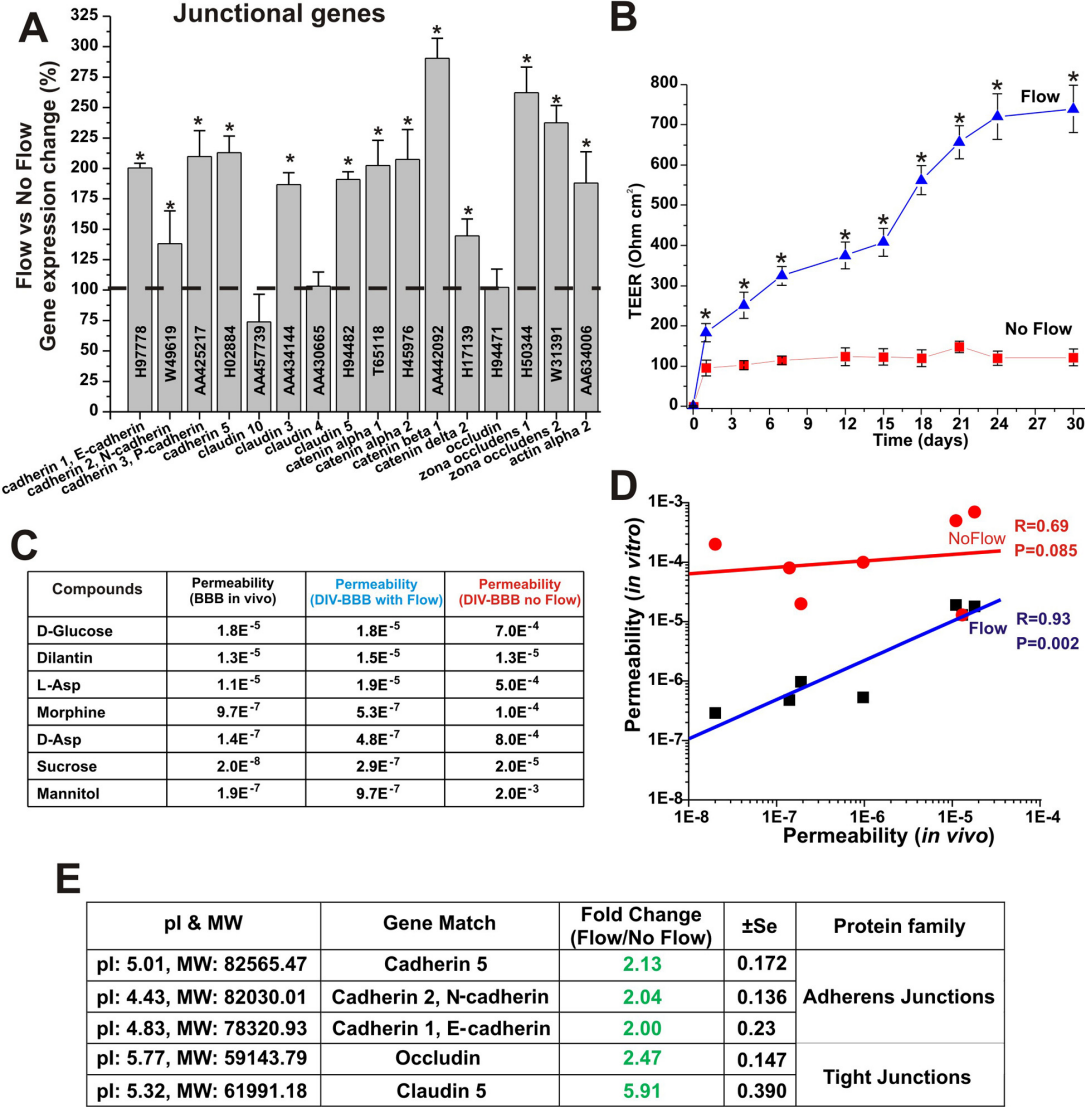
**Laminar shear stress promotes tight junction formation**. Panel **A**: Comparison of the level of TJ and adherens junction RNA in EC grown under dynamic (flow) versus static condition. The tightness of the vascular endothelial bed developed under flow culture condition was much more stringent than that grown under in the absence of luminal flow. This was demonstrated by the TEER (Panel **B**) and permeability measurement to known substances (Panel **C**). Note also that the BBB established under flow was capable of discriminating the passage of substances accordingly to their permeability order more efficiently that the BBB established under static condition (Panel **D**). Gene array findings were also supported by comparative analysis of TJ and adherens junction proteins identified and quantified (comparative analysis of the expression levels) on 2D gels of protein extracted from EC membrane sub-cellular fractions (flow and no-flow conditions; Panel **E**). Note that "*" refers to a statistically significant changes in BBB tightness and EC junctional genes expression caused by the exposure to flow (n = 4; p < 0.05).

Gene array analysis from EC samples grown under static and dynamic conditions in presence of abluminal astrocytes showed (see Figure 1A) that exposure to capillary-like SS levels (≅ 6.2 dynes/cm^2^) [6,7] increases the RNA levels of a variety of tight and adherens junction components such zonula occludens-1 (ZO-1), Claudin 3 and 5, cadherins, catenin α2 and β1, and actin α2 [8]. Actin filaments in conjunction with catenin molecules provide the structural cellular connection with the transmembrane proteins forming the inter-endothelial tight and adherens junctional complexes [9]. The functional significance of this response to SS is quite evident if one looks at the structural integrity and tightness of the corresponding vascular endothelial beds. As shown in Figure 1B EC co-cultured with AH under the influence of SS formed a significantly more stringent barrier (TEER≈700 Ohm cm^2^) than parallel co-cultures grown under static condition (TEER≈100 Ohm cm^2^). Barrier selectivity is significantly affected by the tightness of the vascular bed formed. As shown in Figures 1C and 1D EC grown under static conditions did not form a viable barrier capable of differentiating the passage of substances in relation to their real (*in vivo*) permeability distributions. Results from gene array analysis were supported by comparative protein quantification of tight and adherens junction proteins in 2D gels of endothelial protein extracts from membrane sub-cellular fractions of EC harvested from the co-culture systems. Figure 1E shows the expression ratio (flow vs. no-flow) of key TJ components as well as that of adherens junction protein identified on the 2D gels. Exposure to a physiological capillary level of SS significantly increased the expression level of TJ components occludin (2.47 ± SEM 0.147) and claudin 5 (5.91 ± SEM 0.390). The expression level of the adherens junctions cadherin-1 (2.04 ± SEM 0.136), -2 (2.00 ± SEM 0.230), -5 (2.13 ± SEM 0.172) were also unregulated. Data were expressed as fold changes (p < 0.05).

### Shear stress induces the endothelial expression of drug transporters and metabolic properties that allow the BBB to shield the CNS from potentially harmful substances

**Figure 2:**
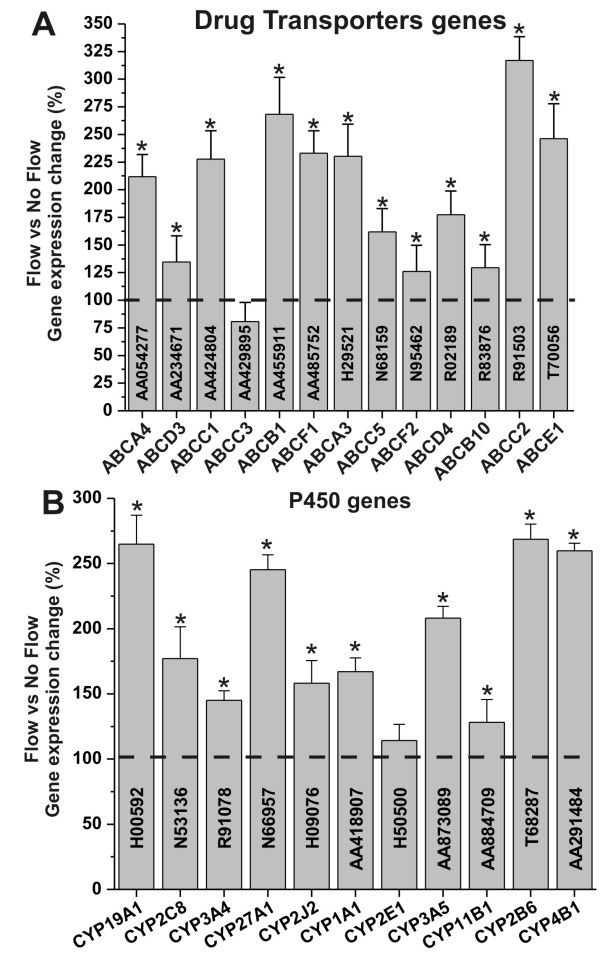
**Shear stress enables vascular-dependent shielding of brain from unwanted and potentially harmful substances**. Shear stress increases the RNA levels of gene encoding for multidrug resistance proteins (panel **A**) and cytochrome P450 enzymes (panel **B**). Note that "*" refers to a statistically significant (n = 4; p < 0.05) increase of the genes transcription in EC exposed to flow versus those grown under static conditions.

Pivotal properties of the BBB are the shielding from potentially harmful substances and the selective permeability to ions and other nutrients/substances. Exposure to physiological levels of SS Increased the RNA level of multidrug resistance transporter (see Figure 2A) commonly expressed at the BBB *in vivo*. These included ABCB1 (MDR1), ABCC1 (MRP1), ABCC2 (MRP2), and ABCC5 (MRP5) [10]. Recent studies have also shown that in addition to multidrug resistance proteins, BBB endothelial cells express a variety of Cytochrome (CYP) P450 enzymes which may synergistically contribute to regulate the passage of substances into the brain [3,11]. We now report that exposure to SS increased the endothelial RNA levels of several CYP450 enzymes (see Figure 2B). These included members of the CYP1, CYP2, and CYP3 families (CYP1A1, CYP2B6, CYP2C8, CYP2J2, CYP3A4, CYP3A5), which are mainly involved with steroid and drug metabolism [3,12]; CYP4B1 is a monooxygenase engaged in the metabolic transformation of fatty acids and the synthesis of cholesterol; CYP11B1 is a steroid 11β-hydroxylase involved in the biosynthesis of steroids. Additional CYP RNA levels that were significantly upregulated are that of CYP19A1 (biosynthesis of estrogens) and that of CYP27A1. The latter is a mitochondrial P-450 enzyme with broad substrate specificity for C27 sterols (e.g., cholesterol degradation).

### Exposure to flow promotes the endothelial expression of ion channels and specialized transport systems

**Figure 3:**
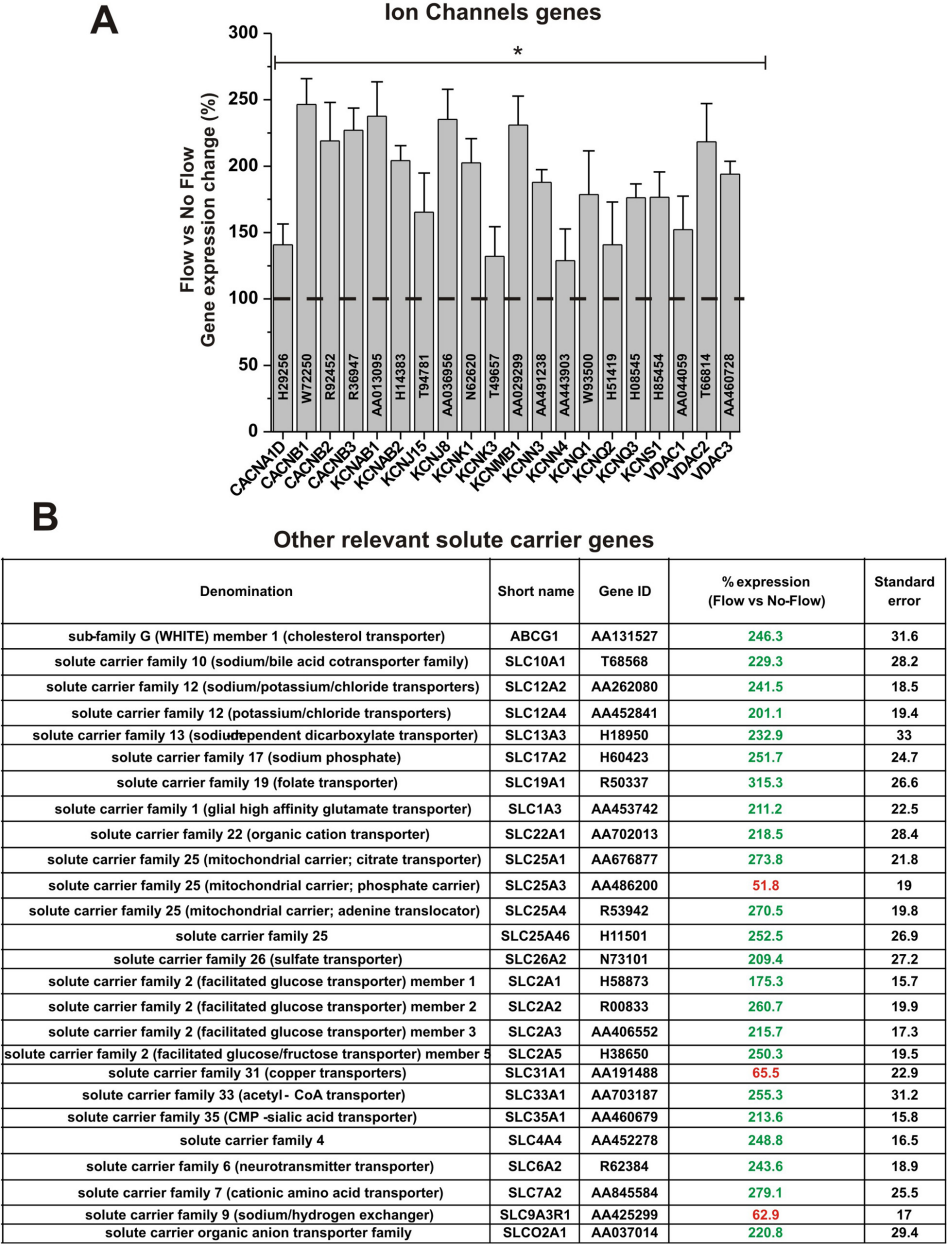
**Shear stress enhances the RNA levels of ion channel genes and other relevant transporters**. Note that "*" refers to a statistically significant (n = 4; p < 0.05) increase of the genes transcription in EC exposed to flow versus those grown under static conditions. Note also that the data in panel **B **are reported as % of the RNA level measured in EC grown under static condition ± SEM.

Our results (see Figure 3A) showed that exposure to flow increased the RNA levels of different classes of ion channels. These included voltage-gated potassium channels (e.g., KCNCB1, KCNCB2, KCNQ1, KCNQ2, and KCNQ3), voltage-dependent calcium channels (e.g. CACNA1D, CACNB1, CACNB2, and CACNB3) inwardly rectifying potassium channel (e.g., KCNJ15) delayed-rectifier potassium channels (e.g., KCNS1) and other voltage-dependent anion channel (VDAC1, 2, and 3).

Among the many roles played by the BBB that of supplying essential nutrients to the CNS and regulating ionic trafficking and fluid movements between the blood and the brain are considered among the most crucial functions. The BBB has a very low permeability to ions and polar molecules thus, the presence of specific transporters is essential to regulate the trafficking of these substances across the vascular endothelium. Our results (see Figure 3B) showed that the RNA level of a number of crucial endothelial transporters was significantly altered by the exposure to physiological capillary levels of SS. RNA level of members of the "facilitate glucose transporters family" such as Glut-1 (SLC2A1), Glut-2 (SLC2A2), Glut-3 (SLC2A3) and Glut-5 (SLC2A5) were significantly increased. That of Acetyl-CoA transporter (SLC33A1) was also significantly upregulated (>250% in comparison to static culture conditions). Similar trend was observed for the RNA level of organic anions and cations transporters (SLCO2A1 and SLC22A1) as well as that of cationic aminoacids (SLC7A2), folate (SLC19A1), sulfate (SLC26A2), mitocondrial carriers (SLC25A1 and SLC25A4). Several ions transporters (SLC12A2. SLC12A4) were also upregulated (> 200% in comparison to static condition). By contrast, the RNA level of sodium/hydrogen exchanger (SLC9A3R1), the copper transporter (SLC31A1), and that of a mitochondrial phosphate carrier (SLC25A3) were decreased. All the results were statistically significant (n = 4; P < 0.05).

### Shear stress facilitates endothelial-leukocyte cross-talk to respond to pro-inflammatory stimuli

**Figure 4:**
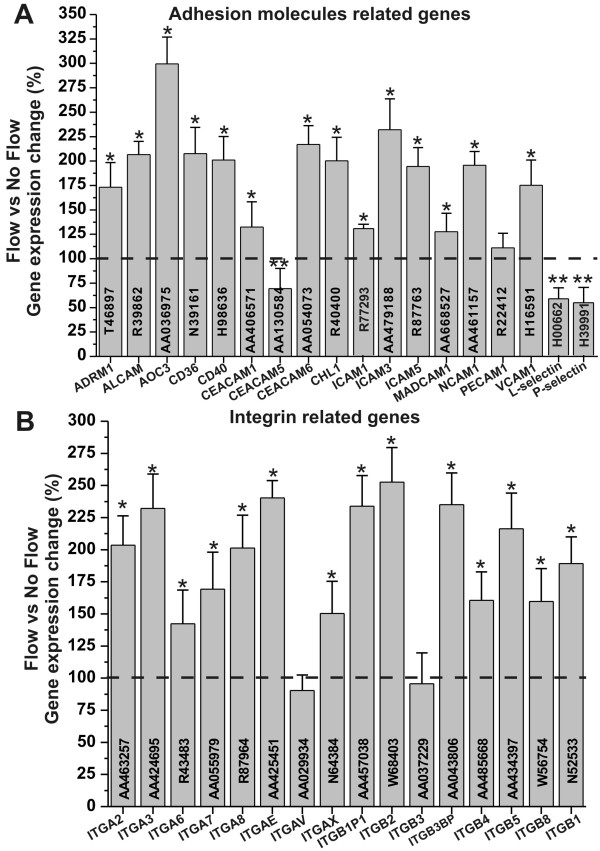
**Shear stress enables endothelial-leukocyte interaction**. Shear stress increased the transcription level of relevant adhesion molecules (Panel **A**) and integrins (Panel **B**) thus facilitating the cross-talk between the vascular endothelium and the host immune system at the BBB level. Note that "*" and "**" refers to a statistically significant (n = 4; p < 0.05) changes (increase and decrease respectively) of adhesion molecules and integrins genes transcription in EC exposed to flow versus those grown under static conditions.

The recruitment of leukocytes from the blood stream and their subsequent interaction (rolling, adhesion and locomotion) with the vascular endothelium are critical stages of the immune response during inflammation. The detail mechanisms controlling leukocyte-endothelial interaction at the BBB as well as the molecular mediators that are involved in the process are not fully understood. It is clear however, that the vascular endothelium plays a crucial role in this process through the presentation of various adhesion ligands at sites of inflammation. Our study (see Figure 4A) has shown that exposure to SS increased the RNA levels of several key adhesion molecules including ICAM1, VCAM1 and PECAM1, which play an important role on the adhesion and migration of T lymphocytes [5,13]. E and P-selectin RNA was instead downregulated. Some data suggest that they do not have a critical role in leukocyte recruitment across the BBB [14] but their function is not fully understood. Integrins play a role in cell signaling and therefore they are involved with the interactions between ECs and the surrounding environment. Exposure to SS increases the RNA level of several integrins (see Figure 4B). For example the alpha chains 2, 3, 7 (ITGA2, ITGA3, ITGA7) and the beta chains 1, 5, and 8 (ITGB1, ITGB5, and ITGB8) interact with the extracellular matrix (ECM) [15].

### Shear stress modulate the bioenergetic behaviour on the BBB endothelial cells

**Figure 5:**
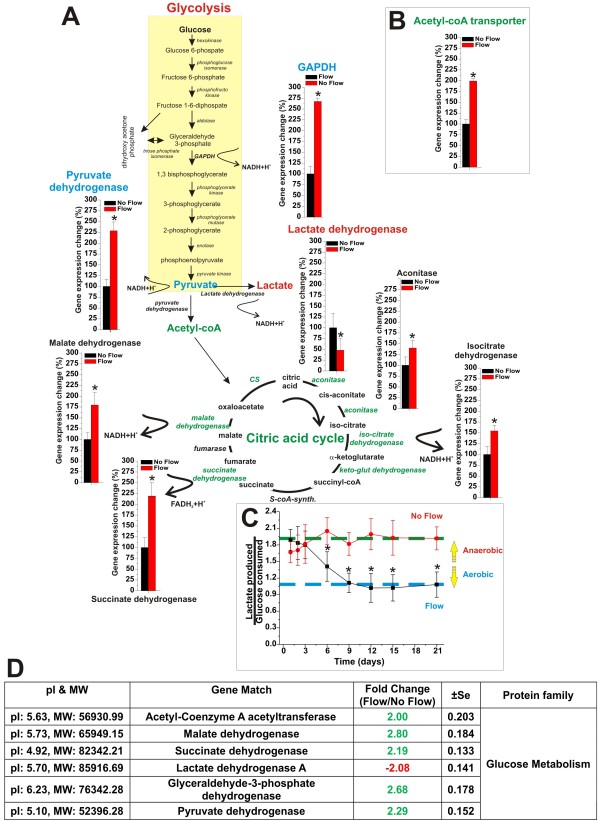
**Shear stress modulates the endothelial bioenergetic pathway**. Panel **A **and **B **Enzimatic pathways of glycolysis and Krebb's cycle. Note the change in the expression of genes encoding for the key regulator enzymes of glucose metabolism and how the exposure to flow greatly favoured the more efficient aerobic pathway. The gene array data were confirmed by measurements of glucose consumption versus lactate production in DIV-BBB model developed with and without l endothelial exposure to intraluminal flow (Panel **C**). Comparative analysis of the expression level of key enzymes regulating the glycolitic and the aerobic (Krebb's cycle) pathways strongly supported the gene array data (Panel **D**). Note that the lactate production/glucose consumption ratio measured in the flow-exposed in vitro BBB modules was ≈ 1. Complete anaerobic metabolism would produce 2 lactates/glucose (ratio = 2) thus, indicating that at least 50% of the glucose consumed underwent aerobic metabolism.

Our results (see Figure 5A) have shown that the ECs RNA level of the key metabolic switch controller (anaerobic to aerobic pathway) pyruvate dehydrogenase is significantly increased by the exposure to flow. By contrast that of lactate dehydrogenase (aerobic to anaerobic pathway) is decreased. In addition to that, the RNA level of many dehydrogenases and other key enzyme controlling the Krebb cycle (aerobic respiration) including the Acetyl-coA transporter (see Figure 5B) were also increased. The effect of flow on the glucose metabolism was confirmed by measurements of glucose consumption and lactate production over a period of 3 weeks from the establishment of the co-culture systems (see Figure 5C). Our experimental data showed that endothelial cells grown under dynamic conditions develop a metabolic behavior that makes significant use of aerobic respiration. This is demonstrated by the comparative measurements of lactate produced over glucose consumed (ratio ≈ 1) showing that at least 50% of the glucose is channeled into the citric acid cycle and metabolically converted in CO_2 _and H_2_O with a significant release of bioenergetic equivalents (ATP; NADH, etc.). This was not observed in parallel co-cultures where ECs were not exposed to flow. In this case we measured a lactate produced over glucose consumed ratio ≈ 2. In this case each molecule of glucose underwent anaerobic metabolic transformation into two molecules of lactate.

These data further strengthen previous funding [16] suggesting that the exposure to intraluminal flow plays a key role in determining the intracellular bioenergetic pathway (aerobic or anaerobic). This hypothesis is also supported by the fact that the corresponding expression levels of the key enzymes controlling the aerobic metabolic pattern were also upregulated (see Figure 5D). By contrast, the expression of lactate dehydrogenase, (which converts pyruvate into lactate thus, ending the glycolytic energy biotransformation of glucose) was downregulated.

### Laminar shear stress inhibits endothelial cell proliferation

**Figure 6:**
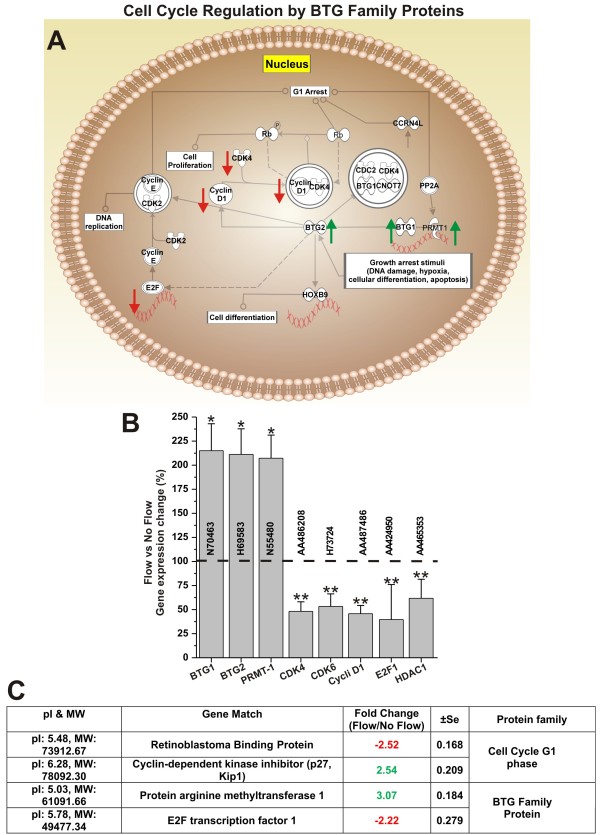
**Shear stress inhibits cell proliferation**. Panel **A**: schematic representation of the cell cycle regulation by BTG family proteins. Functional correlation between gene expression and cell cycle was determined using Pathway analysis software. Note how shear stress increased the RNA levels of genes encoding the cell cycle inhibitory effectors BTG1, BTG2 and PMRT1 (Panel **B**). This finding was also supported by the comparative analysis (flow vs. no-flow) of the expression level of cell cycle modulatory proteins (Panel **C**). Note that genes and protein related to positive modulators of the cell cycle progression were significantly downregulated. Note also that "*" and "**" refers to a statistically significant (n = 4; p < 0.05) changes (increase and decrease respectively).

Inhibition of cell proliferation is a common prodromic event to cell differentiation. Our results showed that SS affects the transcription of genes involved in the cell cycle regulation. BTG proteins are in this specific case novel regulators of transcription engaged in the control of the cell cycle. Although the biological array of functions of BTG proteins is not fully understood, it is known that BTG1 act as a growth arrest gene responsible for the maintenance of the quiescent state, while BTG2 acts as a negative regulator of the cell cycle. Therefore both BTG1 and 2 act as negative regulators of cell proliferation (see Figure 6A). Our results have shown that SS significantly increases the RNA level of both B-cell translocation gene 1 (BTG1) and gene 2 (BTG2) (see Figure 6B). BTG1 and 2 also interact with protein arginine N-methyltransferase 1 (PRMT1) [17], which activity is critical for growth factor-induced cell differentiation [18]. By contrast, the RNA level of key regulators G1/S checkpoint phase which favor cell division such as CDK4/6-cyclin D, Cyclin D1, the transcription factor E2F1 as well as that of histone deacetylase 1 (HDAC1) [19,20] was significantly decreased. Gene array data were also supported by parallel analysis and comparative quantification of the corresponding proteins (see Figure 6C). Our data have shown that SS significantly increased the expression of PRMT1 (3.07 ± SEM 0.184) as well as that of cyclin-dependent kinase inhibitor (p27, Kip1) (2.54 ± SEM 0.209). By contrast, retinoblastoma binding protein and E2F transcription factor 1 were significantly downregulated (-2.52 ± SEM 0.168 and 2.22 ± SEM 0.279 respectively).

**Figure 7:**
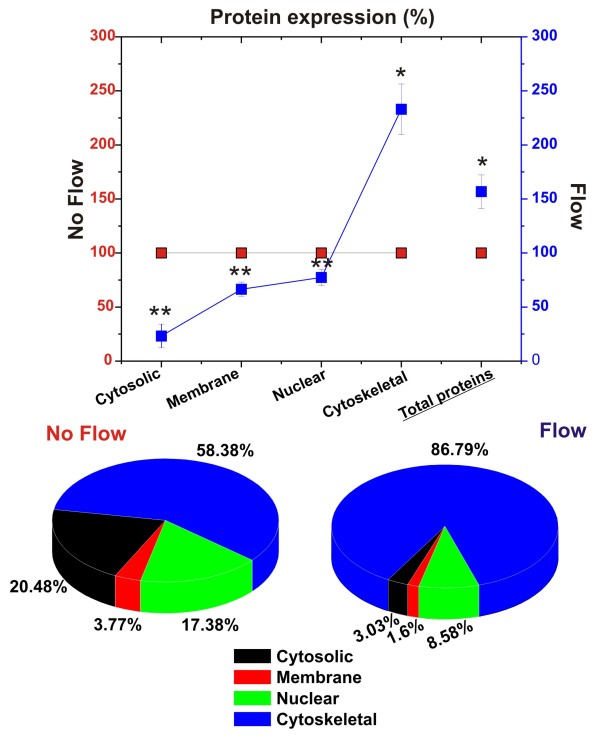
**Shear stress increases cytoskeleton protein expression while decreasing that of cytosol, nucleus and membrane**. Note the drastic changes related to cytoskeletal protein expression. In EC grown under flow condition cytoskeletal proteins represent account for majority of the total protein content. Note also that the exposure to flow significantly decreased the overall expression of nuclear, cytosolic and membrane proteins. This is consistent with endothelial morphologic adaptation to flow and with a reduced cell proliferation. Note that "*" and "**" refers to a statistically significant (n = 4; P < 0.05) changes (increase and decrease respectively) in the protein content.

These results were also indirectly corroborated by measurement of the sub-cellular (nucleus, cytoplasm, cytoskeleton and membrane) and total protein content (see Figure 7). Our results showed that the exposure to flow significantly increased the expression of cytoskeletal proteins (see Figure 7A) while reducing that of the other sub-cellular fractions. The total amount of protein content/cell was increased by flow and the sub-cellular protein content showed that the largest component of the total cellular protein pool (86.79%) was made by cytoskeletal proteins (see Figure 7B) with cytosolic, nuclear and membrane protein pools significantly reduced (20.48 to 3.03%; 17.38 to 8.58%; and 3.77 to 1.6% respectively).

## Discussion

BBB endothelial cells *in vivo *are continuously exposed to laminar shear stress to which they respond by structural (cell orientation with flow direction; redistribution of cell fibers and flattening) and functional remodeling showing significant evidences of differentiation [21-24].

One of the main functions of the BBB is shielding of the brain from unwanted and potentially harmful substances. Tight junction protein complexes provide a mechanical means to seal the paracellular pathways between adjacent endothelial cells [8,24]. Our study shows that exposure to flow increases the RNA levels of genes encoding for a variety of tight junctional proteins (see Figure 1A) including the intracellular scaffold proteins Zonula occludens-1 (ZO1) and 2 (ZO-2) which link the junctional molecules claudin and occludin to intracellular actin and the cytoskeleton. Claudin 3, 5 and actin gene espression were also upregulated by the exposure to laminar shear stress thus strongly suggesting that acting as a key regulator of TJ expression. In addition to TJ protein upregulation we also found a significant increase the RNA and protein levels of several cadherins. These are a class of type-1 transmembrane proteins which play important roles in cell adhesion, through the formation of adherens junctions. Our data showed the up-regulation of N-, P- and VE-cadherin. We also observed an increase in the RNA and protein levels of E-cadherin. However, even though the upregulation of E-cadherin (commonly though not exclusively expressed in epithelial cells [25,26]) was statistically significant; its overall RNA and total protein expression levels were very modest in comparison to that of the other adherens junction components.

The result is the formation of tight barrier with high trans-endothelial electrical resistance that can efficiently discriminate the passage of substances according to their permeability coefficient. In the absence of intraluminal flow despite the presence of abluminal astrocytes the resulting barrier is much less stringent and does not provide selective permeability (Figure 1D). This demonstrates that glial stimuli are important but not sufficient to establish a functional BBB and that exposure to flow provides the missing modulatory element to enable BBB properties.

BBB shielding function however, is also achieved at other levels: 1) by mechanisms of drugs extrusion. This is carried out by ATP-binding cassette transporters (ABC transporters) which are transmembrane proteins that utilize the energy of adenosine triphosphate (ATP) hydrolysis to translocate a broad range of substrates across the cellular membranes [27-29]; 2) by enzymatic metabolism of substances that enter the cell. This function performed is by cytochrome P450 enzymes similar to those present in the hepatocytes. These enzymes can use a variety of molecules (including exogenous substances such as drugs and potentially toxic chemicals) as substrates in enzymatic reactions [3,12].

Our study shows that exposure to flow increases gene expression of several efflux transporters including the multidrug resistance 1 (*MDR1/ABCB1*) gene (MDR1), and members of the multidrug resistance associated protein family MRP1 (ABCC1), MRP2 (ABCC2), and MRP5 (ABCC5) (see Figure 2A). P-glycoprotein (P-gp) the products of the MDR1 gene is an effluxer with specificity for cationic or electrically neutral substrates as well as a broad spectrum of amphiphilic substrates [30] while MRPs are multispecific organic anion efflux transporters. Togheter these efflux transporters can drastically reduce brain penetration of a broad range of endogenous and exogenous substances which do not follow a paracellular pathway to cross the vascular endothelium.

The "third" barrier is instead metabolic in nature. The BBB endothelium is also provided with cytochrome P450 enzymes which catalyze the oxidation of organic substrates such as lipids and steroidal hormones but also exogenous substances including drugs [3].

Recent studies [11] have shown that BBB endothelial p450 enzymes may be co-responsible for the onset of drug resistance to pharmacological treatments. For example, carbamazepine (CBZ) is an anticonvulsant currently used in the pharmacological treatment of epileptic patients. CBZ is a P450 CYP3A4 substrate. Overexpression of CYP3A4 has been shown to correlate with that of MDR1 at the blood-brain barrier interface of patients with drug refractory epilepsy. Our study has shown that exposure to flow significantly increases gene expression levels of most members of the cytochrome P450 families CYP1 (including CYP3A4), CYP2, and CYP3. Taken togheter the increased gene expression of multidrug resistance proteins and P450 enzymes provides a complex mechanism to regulate the CNS drug bioavailability, or to shield the CNS.

The RNA level of CYP11B1, and CYP19A1 were also upregulated by flow. These enzymes are involved in the biosynthesis of steroids and estrogen, which modulate many biological activities including BBB permeability and leukocytes adhesion [31,32]. CYP27A1 RNA level was also significantly upregulated by flow. These P450 enzymes are responsible for the degradation of cholesterol and play an in important role in the maintenance of cholesterol pool in the CNS [33,34]. This is in agreement with the fact that enhancement of endothelial permeability and lipid deposition preferentially occurs at turbulent, low shear stress regions such as in the arteries [35].

The shielding provided by the BBB may on the other end limit the paracellular diffusion of polar substances including ions and essential nutrients (e.g.,glucose and amino acids) necessary for the maintenance of neuronal activity. Therefore the BBB endothelium must be equipped with specific transporters to supply the CNS with these substances without compromising its barrier functions and its ability to maintain the homeostasis of the brain environment. Our data have shown that the exposure to SS positively modulates the translational expression of Ca^2+ ^and K^+ ^ion channels. Calcium and potassium homeostasis play an important role in the control of neuronal excitability but are also involved in macrophage transmigration across the vascular endothelium at the BBB [36]. Furthermore, Ca^2+ ^is involved in the modulation of BBB integrity and endothelial morphology [37,38].

In addition to ion channels the gene expression level of other specialized solute carriers was also found to be modulated by flow. These include members of the glucose transporters family Glut-1, Glut-2, Glut-3 and Glut-5 [39,40]; solute carriers specific for cationic aminoacids, organic anions and cations, folate, sulfate as well as the Na-K-Cl symporter and the potassium-chloride cotransporter. All together these specialized functions represent the hallmark of a drastic differentiation process that set the brain microcapillary endothelium aside from those forming other vascular beds.

Transporters allow nutrients and other biologically important substances to transit from the blood into the brain across the BBB. To efficiently meet the bioenergetic demand of the many active transport systems that are critical for sustaining neuronal function the BBB ECs make a substantial use of aerobic respiration or Citric acid cycle. This is an oxigen-dependent 8-step enzimatic process that allows the full conversion of 2 molecules of acetyl-CoA (derived from the conversion of glucose during glycolysis) into carbon dioxide (CO_2_) and water (H_2_O). The theoretical energetic yield of the process from the complete oxidation of one glucose molecule to CO_2 _and H_2_O is of 6 NADH, 2 FADH_2_, and 2 ATP (equivalent to 36 ATP molecules). The aerobic pathway also provides reductant equivalents (such as NADH) to counteract the harmful effect of reactive oxidative species generated intracellularly or originating by exposure to xenobiotic substances.

Our results show that exposure to flow plays a critical role in the modulation of the BBB-Ec bioenergitic metabolism favoring the expression of genes encoding for the key enzymes operating the conversion of acetyl-CoA into CO_2 _and H_2_O during the citric acid cycle. Furthermore, shear stress increases the RNA levels of pyruvate dehydrogenase (which converts pyruvate into acetyl-CoA; that of the mitocondrial acetyl-CoA transporter and downregulates the expression level of lactate dehydrogenase which converts pyruvate into lactate (see Figure 5A, B and 5D). The aerobic metabolic induction operated by flow on the BBB endothelium was further confirmed by lactate production and glucose consumption measurements demonstrating that at least 50% of the glucose was processed by aerobic respiration.

An important implication of this "metabolic modulation by flow" is that BBB endothelial cells can adapt their metabolic process according to the availability of oxygen. This is dependent on blood flow therefore, It is logic to postulate that whether blood flow is interrupted (e.g., ischemia or stroke) or the oxygen content in the blood is reduced (venules), the vascular endothelium can switch to an anaerobic respiration to meet its energetic demand.

Controls on cell proliferation and differentiation are interrelated even though it is still not clear to what extent. Our data (see Figure 6) show that exposure to flow increases the RNA levels of BTG proteins (BTG1 and 2) which are negative regulators of the cell cycle [41]. BTG1 or BTG2 bound to PRMT 1 (which RNA and protein expression levels were also increased by flow; see Figure 6B and 6C) is required to induce growth inhibition [17]. Furthermore, the RNA level of cyclin D, cdk4, and 6, which control the re-entry of resting G0 cells into the G1 phase of cell cycle were downregulated. Cells that exit the cell cycle to enter and stay at resting (G0) phase is characteristic of terminally differentiated cells. Quantitative analysis of the protein content (see Figure 7) relative to nucleus, cytosol, cytoskeleton, and membrane sub-cellular fractions have shown a drastic reduction of the nuclear protein content in EC exposed to flow. This is in agreement with the lack of DNA synthesis in resting cells and the fact that in vivo the endothelial DNA synthesis preferentially occurs at branch orifices characterized by low and turbulent flow [42]. Furthermore, we observed a substantial increase in cytoskeletal protein content. This can be related to a morphological adaptation of the BBB endothelium to flow and the necessity to provide structural support for TJ protein complexes [24].

Taken togheter our results suggest that the exposure to a capillary level laminar shear stress on one hand favors EC differentiation into a BBB phenotype and on the other hand inhibits the cell cycle. However, whether the two processes are simultaneously regulated is still unclear.

## Conclusion

Shear stress plays a key role in modulating endothelial structure and function. Our study shows that SS-dependent effects on EC are modulated by induction/suppression of genes regulating multiple aspects of endothelial physiology; from the formation of inter-endothelial tight junction to the expression of specific carrier-mediated transporters and drug resistance mechanisms. Studies by other have also shown that SS induces the production of vasoactive substances [43]. Turbulent, but not laminar shear stress stimulates EC turnover [44]. Accordingly, *in vivo *DNA synthesis of EC preferentially occurs at branch orifices with low flow rates [45,46] which is paralleled by the enhancement of endothelial permeability [47]. Genomic and proteomic analyses are currently being used to study the BBB and how it relates to the pathogenesis of major neurological diseases [48]. In this respect this study provides the bases for new hypotheses and future studies to unveil novel unexploited clinical targets that can facilitate the development of innovative therapeutic strategies to reduce the burden of BBB-related CNS diseases.

## Methods

### Cell Culture

Normal human brain microvascular endothelial cells (HBMEC, cat# 1000, ScienCell Research Laboratories, San Diego, CA 92121) and human astrocytes (HA, cat# 1800) were initially expanded in 75 cm^2 ^flasks pre-coated with fibronectin (3 μg/cm^2^) and Poly-d-Lysine (3 μg/cm^2^) respectively. Endothelial growth medium consisting of MCDB 105 (Sigma, Cat# M6395), 10% human AB serum (SIGMA, Cat# S-7148), 15 mg/100 ml of endothelial cell growth supplement (ECGS, Cat.# 1052), 800 units/ml of heparin (Sigma, cat# H3393), 100 units/ml penicillin G sodium and 100 mcg/ml streptomycin sulfate was used to expand HBMEC cultures. HA were expanded in Dulbecco's modified essential medium (DMEM-F12) supplemented with 2 mM glutamine, 5% fetal bovine serum (FBS), 100 units of penicillin G sodium per ml, and 100 μg of streptomycin sulfate per ml. HBMEC and HA were maintained at 37°C in a humidified atmosphere with 5% CO_2_. Cellular growth was monitored every day by inspection with phase contrast microscopy. To minimize the dedifferentiation process cell cultures were not expanded for more than two cycles. Prior to use at the end of the expansion process HBMEC were characterized by immunocytochemistry using sheep polyclonal antibodies that recognized the human Von Willebrand Factor Antigen VIII (vWF/Factor VIII, US biological, Swampscott, MA, cat# F0016-13A) and were found to be > 99% pure endothelial cells [49]

### DIV-BBB setup

HBMEC and HA were cultured in the DIV-BBB, as previously described [50,51]. Briefly, HBMEC are first inoculated into the luminal compartment and allowed to adhere under static conditions over a 48-hr period. In order to achieve higher levels of cell attachment, the flow path was canalized through the extra-capillary space. Endothelial cells were then exposed to a low-level shear stress (1 dyne/cm^2^) for 24 hours. The shear stress was then raised to a constant value of 4 dyne/cm^2 ^over the course of the following week and then brought to final (capillary-like) level of 6.2 dyne/cm^2^[6,7]. Please note that our current pumping system allows us to generate within the DIV-BBB system shear stress levels ranging from 0.8 up to 42 dyne/cm^2^. Astrocytes were seeded on the abluminal surface of the fibers three days after the initial loading of the endothelial cells. Typically two weeks of co-culture are required to establish a fully functional BBB with a TEER greater than 600 Ω cm^2 ^above the baseline (empty module).

To maintain similar culture conditions without exposing the endothelial cell (EC) to flow, parallel DIV-BBB modules were established with the following procedure. Astrocytes were first seeded in the abluminal space and left adhere for 48 hours while maintaining a luminal medium flow. Then the flow path was canalized through the extra-capillary space and the endothelial cells were seeded intraluminally. Flow was then maintained extraluminally for the duration of the experiment.

At the end of the experiment EC were harvested and processed for RNA purification and protein extraction from the cytosol, membranes, nucleus, and cytoskeleton using Qproteome Cell Compartment Kit (Quiagen, Valencia, CA cat# 37502) according to the protocol provided by the manufacturer.

### TEER measurement

BBB formation was monitored by real time measurements of trans-endothelial electrical resistance (TEER; Flocel Inc., Cleveland OH). The device interfaces directly to a PC computer via Universal Serial Bus (USB) and utilizes electronic multiplexing to assess the integrity and viability of tissue culture bilayers. The microcontroller computes the resistivity and capacitance per cm^2 ^of the barrier from physical parameters. TEER was measured continuously from the initial setup throughout the course of each experiment. Previous work in our laboratory [16] showed a direct (inverse) relationship between TEER and BBB permeability in the DIV-BBB.

### Glucose consumption and lactate production

Medium samples were simultaneously collected every few days from the luminal and abluminal compartments of each DIV module. The concentration of glucose and lactate was determined via a dual channel immobilized oxidase enzyme analyzer (YSI 2700 SELECT, YSI Inc., Yellow Springs, OH). Samples were kept frozen after being collected and processed at a later time, when multiple samples from the same experiment could be run simultaneously. The enzyme analyzer was set to recalibrate itself six samples as multiple samples were analyzed in the same run.

### RNA Extraction

Endothelial cells were purged from the DIV modules by gentle enzymatic dissociation using trypsin and 2 mg/ml collagenase and collected by centrifugation. Note that EC were compartmentally isolated from the abluminal astrocytes by the hollow fibers wall which is not permissive for intercompartimental cell migration across. EC from suspension were pelleted by centrifugation at 1,000 × g. Supernatant media was removed without washing and cells were incubated in TRIZOL Reagent (1 ml/5-10 ×10^6 ^cells) for 5 minutes at 30°C to permit the complete dissociation of nucleoprotein complexes. Next, 0.2 ml of chloroform/ml of TRIZOL were added. Sample tubes were capped and shaken vigorously 15 seconds and left to incubated at 30°C for 2 to 3 minutes. Samples were then centrifuged at 12,000 ×g for 15 minutes at 8°C. Following centrifugation, the mixture separated into a lower red, phenol-chloroform phase, an interphase, and a colorless upper aqueous phase containing the RNA.

### RNA precipitation

RNA from the aqueous phase was precipitated by mixing with 0.5 ml of isopropyl alcohol/1 ml of TRIZOL and left to incubate for 10 minutes at 30°C following by centrifugation at 12,000 ×g for 10 minutes at 8°C. The supernatant layer was then removed and the RNA pellet was washed, once with 75% ethanol (1 ml/1 ml of TRIZOL used for the initial homogenization). The samples were mixed by vortexing and centrifuged at 7,500 ×g for 5 minutes at 8°C. The RNA pellet was then dissolved in RNase-free water.

### RNA purification and gene analysis

RNA samples were run through Qiagen RNeasy columns (Qiagen USA, Valencia, CA) to remove any genomic DNA contamination. The purity of RNA was determined by measuring the absorbance of the sample diluted in a solution of 10 mM Tris Cl, pH 7.5 buffer at the wavelengths of 260 nm and 280 nm and calculating a ratio of these absorbance values. Pure RNA has an A260/A280 ratio of >1.90. Samples were then processed by Illumina bead-array based gene expression platform at Genomics Core Facility using HumanRefSeq8 BeadChips. Each of these microarrays on the Human-8 Chip contains 23,000 oligonucleotide probes (corresponding to over 23,000 well-characterized genes). Transcription changes were analyzed with Ingenuity Pathway Analysis (IPA^® ^8.7) software (Ingenuity™Systems, Redwood City, CA). This application allows analyzing targeted information on genes, proteins, chemicals, and drugs, and building interactive models of the experimental systems (Path Designer).

Note that genes were considered expressed in the specific population and included in the analysis only if they were flagged as present in all 4 replicates/experimental condition by the gene chip operating software, if average normalized signal intensity minus background value was above a cut off of 25 (according to the manufacturer protocol), and p values were <0.05.

### Endothelial cells protein separation

Protein extraction from the cytosol, membranes, nucleus, and cytoskeleton was performed by using Qproteome Cell Compartment Kit (Quiagen, Valencia, CA cat# 37502) according to the manufacturer protocol for subcellular extraction of proteins. The stepwise extraction delivers four distinct protein fractions from one sample: Cytosolic, membrane/organelle protein, nucleic and cytoskeletal. Proteins are obtained in the native state and readily usable for 2D gel electrophoresis.

### 2D gel electrophoresis

2-D electrophoresis is commonly used to analyze a large number of proteins/sample which can be detected, analyzed and quantified by different means. The proteins isolated as described above were then precipitated with acetone and reconstituted in an isoelectric focusing buffer consisting of 6M urea, 2M thiourea, 2% of 3-(3-chloramidopropyl)dimethylammonio-1-propane sulfate (Chaps), 1% Triton X100, 1% ampholytes and 50 mM dithiothreitol (DTT). Isoelectric focusing was carried out for 43 kVh at 20°C in 11-cm immobilized pH gradient strips (BioRad, Hercules - CA) covering pI 5 to 8, which were loaded with 500 μg total protein using the active rehydration method. The second dimension was carried out in precast 12% SDS-Page gels (Criterion gels, BioRad). Gels were subsequently fixed and stained with Coomassie blue (GelCode Blue, Pierce, Rockford - IL).

### 2D gel analysis

Relative expression of the recovered proteins (flow versus no flow) was determined by densitometric analysis. Two-dimensional protein gels were scanned using a 35-mm camera mounted on a gel scanning unit interfaced to a PC using GelPro™ analysis software. The scanned grayscale images were saved in an uncompressed TIFF format and further analyzed using software specifically designed for measuring grayscale image density, developed by Nonlinear USA, Inc. (Durham, NC) PhoretixTM ID (Version 2003.01).

### Statistical analysis

For parametric variables (e.g., TEER levels, glucose consumption, lactate production, cytokines, and gene expression levels), differences between populations were analyzed by ANOVA. p values <0.05 were considered statistically significant. Bonferroni analysis was used to account for comparisons of multiple parameters among groups. For non-parametric indices (e.g. densitometries for 2D gel electrophoresis), we used Kruskal-Wallis test followed by Mann-Whitney U-test. Endothelial cells isolated from a group of 4 independent DIV-BBB modules per experimental condition were used for the gene analysis, thus the reported gene expression levels are an average of 4 independent arrays. Based upon previous experiments, the number of independent gene arrays replicates analyzed for each experimental condition provides sufficient power to demonstrate statistical significance.

### Disclosure/conflict of interest

Dr. Janigro has reported the following financial relationships with the companies listed below which may be perceived to bias this work.

**Royalty Payments**. Dr. Janigro has the right to receive royalty payments for inventions or discoveries related to Flocel Inc,

**Equity**. Dr. Janigro owns stocks in Flocel Inc.

Dr. Cucullo has reported the following financial relationships with the companies listed below.

**Equity**. Dr. Cucullo owns stocks in Flocel Inc.

Dr. Marchi has reported the following financial relationships with the companies listed below.

**Equity**. Dr. Marchi owns stocks in Flocel Inc.

## Authors' contributions

LC conceived the study, elaborated its design and supervised the project. LC also performed the statistical and pathway analysis and drafted the manuscript. MH carried out the cell culture experiments as well as RNA and protein extraction and purification for gene array analysis. VP carried out the data analysis and protein quantification. NM was responsible for the establishment and maintenance of the tissue culture systems. DJ co-supervised the project and participated in the data analysis. All authors read and approved the final manuscript.
